# Social odors conveying dominance and reproductive information induce rapid physiological and neuromolecular changes in a cichlid fish

**DOI:** 10.1186/s12864-015-1255-4

**Published:** 2015-02-22

**Authors:** José M Simões, Eduardo N Barata, Rayna M Harris, Lauren A O’Connell, Hans A Hofmann, Rui F Oliveira

**Affiliations:** Unidade de Investigação em Eco-Etologia, ISPA - Instituto Universitário, Rua Jardim do Tabaco 34, 1149-041 Lisbon, Portugal; Integrative Behavioural Biology Lab, Instituto Gulbenkian de Ciência, Oeiras, Portugal; Champalimaud Neuroscience Programme, Champalimaud Foundation, Lisbon, Portugal; CCMAR-CIMAR Laboratório Associado, Universidade do Algarve, Campus de Gambelas, 8005-139 Faro, Portugal; Departamento de Biologia, Universidade de Évora, Apartado 94, 7002-554 Évora, Portugal; Institute for Cellular and Molecular Biology, University of Texas at Austin, Austin, TX USA; Department of Integrative Biology, University of Texas at Austin, Austin, TX USA; Institute for Neuroscience, University of Texas at Austin, Austin, TX USA; Current address: FAS Center for Systems Biology, Harvard University, Cambridge, MA USA

**Keywords:** Cichlid, Olfaction, Olfactory bulb, Telencephalon, Microarray, Transcriptomics

## Abstract

**Background:**

Social plasticity is a pervasive feature of animal behavior. Animals adjust the expression of their social behavior to the daily changes in social life and to transitions between life-history stages, and this ability has an impact in their Darwinian fitness. This behavioral plasticity may be achieved either by rewiring or by biochemically switching nodes of the neural network underlying social behavior in response to perceived social information. Independent of the proximate mechanisms, at the neuromolecular level social plasticity relies on the regulation of gene expression, such that different neurogenomic states emerge in response to different social stimuli and the switches between states are orchestrated by signaling pathways that interface the social environment and the genotype. Here, we test this hypothesis by characterizing the changes in the brain profile of gene expression in response to social odors in the Mozambique Tilapia, *Oreochromis mossambicus*. This species has a rich repertoire of social behaviors during which both visual and chemical information are conveyed to conspecifics. Specifically, dominant males increase their urination frequency during agonist encounters and during courtship to convey chemical information reflecting their dominance status.

**Results:**

We recorded electro-olfactograms to test the extent to which the olfactory epithelium can discriminate between olfactory information from dominant and subordinate males as well as from pre- and post-spawning females. We then performed a genome-scale gene expression analysis of the olfactory bulb and the olfactory cortex homolog in order to identify the neuromolecular systems involved in processing these social stimuli.

**Conclusions:**

Our results show that different olfactory stimuli from conspecifics’ have a major impact in the brain transcriptome, with different chemical social cues eliciting specific patterns of gene expression in the brain. These results confirm the role of rapid changes in gene expression in the brain as a genomic mechanism underlying behavioral plasticity and reinforce the idea of an extensive transcriptional plasticity of cichlid genomes, especially in response to rapid changes in their social environment.

**Electronic supplementary material:**

The online version of this article (doi:10.1186/s12864-015-1255-4) contains supplementary material, which is available to authorized users.

## Background

Group living animals have to adjust the expression of social behavior to the nuances of daily social life and to transitions between life-history stages, and their ability to do so impacts on their Darwinian fitness [1]. This socially driven behavioral plasticity induces changes in brain neurogenomic states that underlie different behavioral repertoires [2]. Thus, reprogramming the transcriptome in response to the social environment allows an animal to switch between adaptive behavioral states [3,4]. Gene expression profiling enables the study of this dynamic relationship between genotype and behavior [5] and to unveil the genetic networks behind complex behaviors. In addition, the development of whole-genome sequencing, microarrays and other genomic resources for non-traditional model organisms, but with complex social repertoires, has provided relevant insights on how complex genotypes are translated to produce meaningful behaviors [6-8].

In recent years, numerous studies have described the influence of social environment and of social interactions on transcriptional and neural activity [6]. For example, caste differentiation (between workers/queen) in the honey bee (*Apis mellifera*), a key feature in eusocial insects, is influenced not only by heritable traits but also by variations in the regulation of molecular pathways linked with several life-history traits, such as nutrition, metabolism, and reproduction [9,10]. The activity of aggression-related genes in this species also seems to be under both inherited and environmental influences, varying with age, exposure to alarm-cues and depending on colony environment [11]. The study of gene expression signatures of life history transitions has also been a focus in teleost fishes. For example, life history traits of salmonids have also been addressed in a number of studies showing variation in brain expression profiles related with alternative reproductive and migratory tactics [12,13] and their interaction with the rearing environment [14]. All the results on the impact of the social environment on the transcriptome highlight new possibilities concerning how social stimuli, as well as more complex interactions between conspecifics, can influence and shape gene translation into producing appropriate behavioral responses, according to external and internal cues and also to the animals’ past experience.

Most of the studies discussed above characterize fixed and irreversible behavioral phenotypes, which correspond to switches between “static” neurogenomic states. But the interaction between the genome and the environment is also expected to be present in shorter time frames and to be reversible in order to accommodate labile and transient changes in behavioral states in order for flexible adaptive behavior to evolve [2,15]. Behaviorally, a single interaction may have consequences for the performance of the individuals and the outcome of future interactions (e.g. winner and loser effects of agonistic interactions, [16]; female mate choice, [17]), but its impact on the neurogenomic state of the individuals has been scarcely characterized.

Animals integrate sensory information with internal physiology into context-appropriate behavior that ultimately promotes fitness. Yet how the brain integrates different sensory modalities in these social contexts remains unclear. For example, [18] presented males of the model cichlid *Astatotilapia burtoni* with sensory information in three social contexts: intruder challenge, reproductive opportunity and a socially neutral situation. The authors found that, compared to the neutral context, a visual stimulus was necessary and sufficient for an aggressive response, whereas chemical and visual stimuli presented alone were sufficient for an androgen response. Interestingly, the immediate-early gene c-Fos, a neural activity marker, was induced in response to a visual challenge stimulus specifically in dopaminergic neurons of area Vc (the central region of the ventral telencephalon), a putative striatal homologue, whereas presentation of a chemical stimulus alone did not induce c-Fos expression in the intruder challenge context. Clearly, these results suggest that socially salient sensory cues are processed in a modality-dependent manner in the brain. However, this study did not examine neuromolecular responses of forebrain regions associated with olfactory processing, such as the olfactory bulb or area Dp (posterior portion of the dorsal telencephalon, the putative homolog of the mammalian olfactory cortex (see [19] for a review on the teleost olfactory system).

The Mozambique tilapia, *Oreochromis mossambicus*, is an African cichlid fish that has become a model system in the study of neuroendocrine mechanisms underlying socially mediated behavioral changes (for a review see [1]). The importance of chemical signaling of male social status has been described in this species (e.g. [20,21]), and the olfactory system, from sensory epithelium to bulbar and extrabulbar projections, has been well characterized [22]. In nature, *O. mossambicus* males establish contiguous display territories, which females visit in order to obtain matings*.* The repertoire of social behavior is highly complex and multimodal, including visual (e.g. [23], acoustic [24], and chemical signals (e.g. [20,21]). Importantly, male tilapia store urine in their bladders which they use to signal social rank during agonistic interactions with other males or in the presence of pre-ovulatory females [21]. Furthermore, males are able to modulate their rate of urination depending on the social environment. An increase of males’ urination rate is observed during agonistic encounters [21] or in the presence of pre-ovulatory females [20]. Furthermore, both the volume of stored urine and its olfactory potency, as measured by electro-olfactogram (EOG) recordings, is higher in DOM than in SUB males [20,21]. Females do not store urine and have a higher frequency of urination [25,26]. Additionally, females have smaller kidneys, smaller urinary bladders and the urothelial thickness of the inner surface of the bladder is also smaller than in males [25]. Finally, the odor of pre-ovulatory females elicits higher amplitude EOG responses in males than that of post-ovulatory females [26].

Depending on the social environment, tilapia males can exhibit two distinct behavioral phenotypes: dominants (DOM) and subordinate (SUB). DOM individuals adopt a typical velvet black coloration and establish breeding territories on the bottom, where they dig nests to which they attract females using courtship displays [27,28]. SUB males present a pale silver coloration and either move around among the breeding territories of DOM males or shoal together with females, while they wait for their opportunity for social ascension. Sneaking fertilization attempts by SUB males have also been reported [28]. Changes between these social phenotypes have been shown to activate a cascade of molecular processes and a variety of neuroendocrine pathways which include neuropeptides and steroid hormones [27,29,30]. Ovulated females visit male breeding arenas when ready to spawn and follow courting males to their nests, engage in courtship rituals, and collect the fertilized eggs into their mouths. After spawning, females leave the male aggregations and live in nursery areas located in shallow water while they incubate the eggs in their mouth and care for the fry [31,32]. During this period, females become also more aggressive, defending the brood against predators and conspecifics [33].

Combining physiological with genomic approaches promises to provide novel insights into how simple social signals in a single sensory channel (olfaction) are processed in order to generate context-appropriate behavioral responses. In the present study we therefore first characterize (in DOM males) electrophysiological responses of the olfactory epithelium to different social odors that convey specific information about male social status (DOM vs. SUB) and female reproductive state (pre-ovulatory, PRE vs. post-ovulatory, POST). We then used a 19 K cichlid microarray platform to analyze the corresponding gene expression profiles in the same individuals in specific brain areas known to be involved in the processing of olfactory information: the olfactory bulb and area Dp (olfactory pallium). Our results show that the olfactory system clearly discriminates stimuli depending on social salience physiologically at the sensory periphery and transcriptionally in central processing centers.

## Results and discussion

### Olfactory stimulation

**Figure 1:**
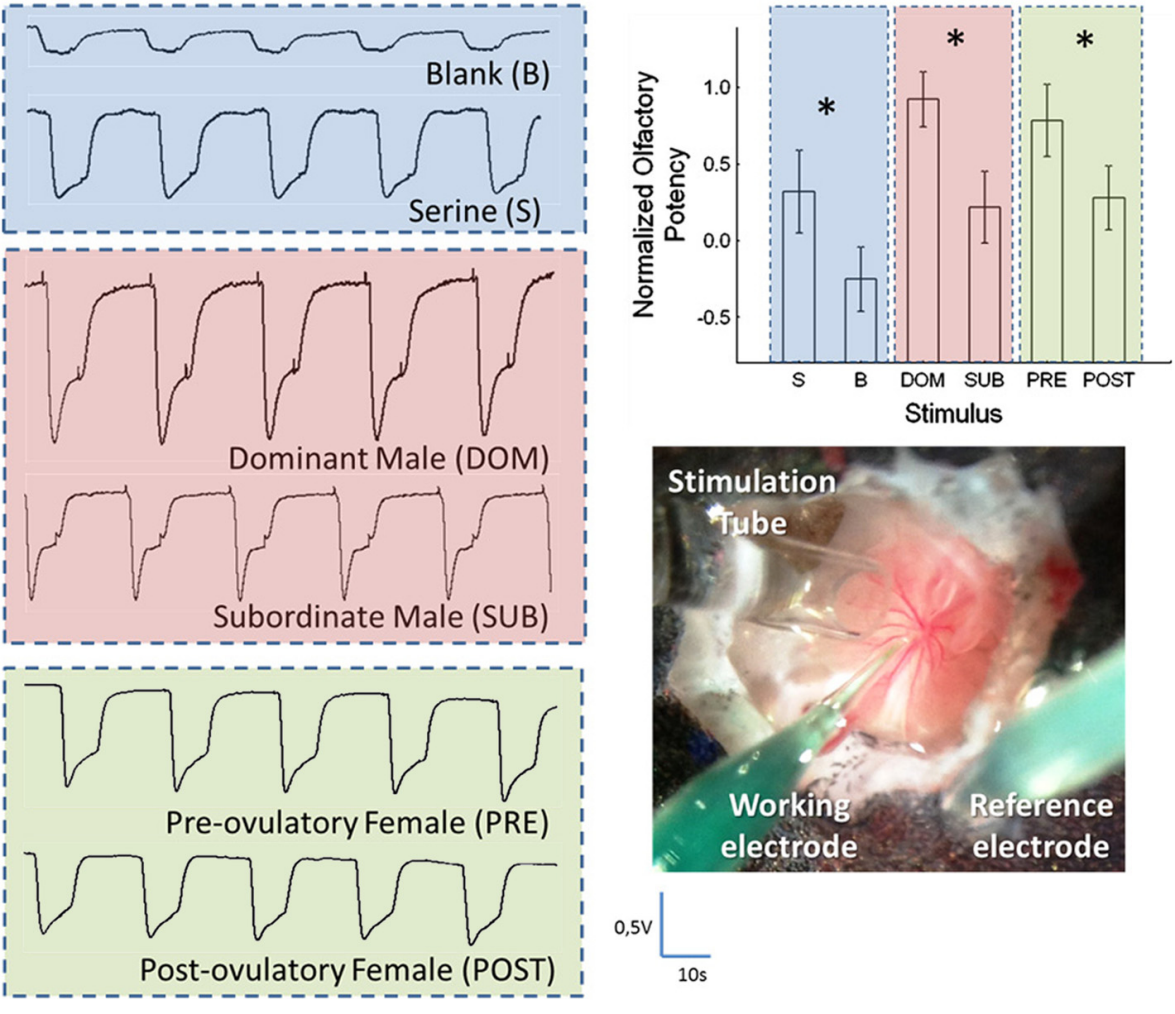
**Olfactory responses of male tilapia to different stimuli.** On the left hand-side, typical electro-olfactograms (EOGs) recorded in response to different stimuli: in blue – controls for normalization – serine (S) and blank (B); in pink – male urine (1:10000) – from dominant (DOM) and subordinate (SUB) males; in light green – extracts of female water (1:1000) – from pre-ovulatory (PRE) and post-ovulatory (POST) females. On the top-half on right hand side, normalized EOG amplitudes (mean ± SEM) elicited by all stimuli: S (N = 6); B (N = 7); DOM (N = 7); SUB (N = 7); PRE (N = 6); POST (N = 6); after 45 min of stimulation (*P < 0.05). On the bottom-half, a depiction of the tilapia’s olfactory rosette (40x) and the apparatus for olfactory stimulation and electrophysiological recording of olfactory evoked potentials.

The overall patterns of response to social odors measured with EOG recordings (Figure 1) were similar to those previously reported for this species [20,21,26]. The mean normalized EOG amplitude evoked by subordinate male urine at a dilution of 1:10000 was significantly smaller (0.25 ± 0.06; N = 7) than that elicited by urine samples of dominant males (0.93 ± 0.10; N = 7; P < 0.01; Figure 1). Furthermore, the mean of normalized responses to water extracts from PRE females at a dilution of 1:1000 (0.79 ± 0.13; N = 6) was significantly higher than that from POST females (0.28 ± 0.10; N = 6, P < 0.01; Figure 1).

Our results show that DOM and PRE stimuli elicited greater responses than SUB or POST stimuli, suggesting that males can discriminate social status and reproductive state of social partners based on olfactory cues alone. The chemical nature of the active odorants which allow for these discriminations is still unknown. Nonetheless, recent work suggests that males can assess a rival’s fighting ability based on the olfactory information present in their urine [25], which might enable them to avoid time consuming and energetically costly escalated fights [34] and thus stabilize social hierarchies [25]. Thus, the EOG responses measured in the sensory neurons at the olfactory rosette suggest that they are well adapted to discriminate between urinary odorants of different male social status, which might contribute to reduce aggression and escalation of fights in a social context. Moreover, males seem to be able to discriminate between females in different stages of their reproductive cycle, probably due to specific odorants released into the water by PRE females, as previously suggested for this species [26].

### Analysis of gene expression profiles

**Figure 2:**
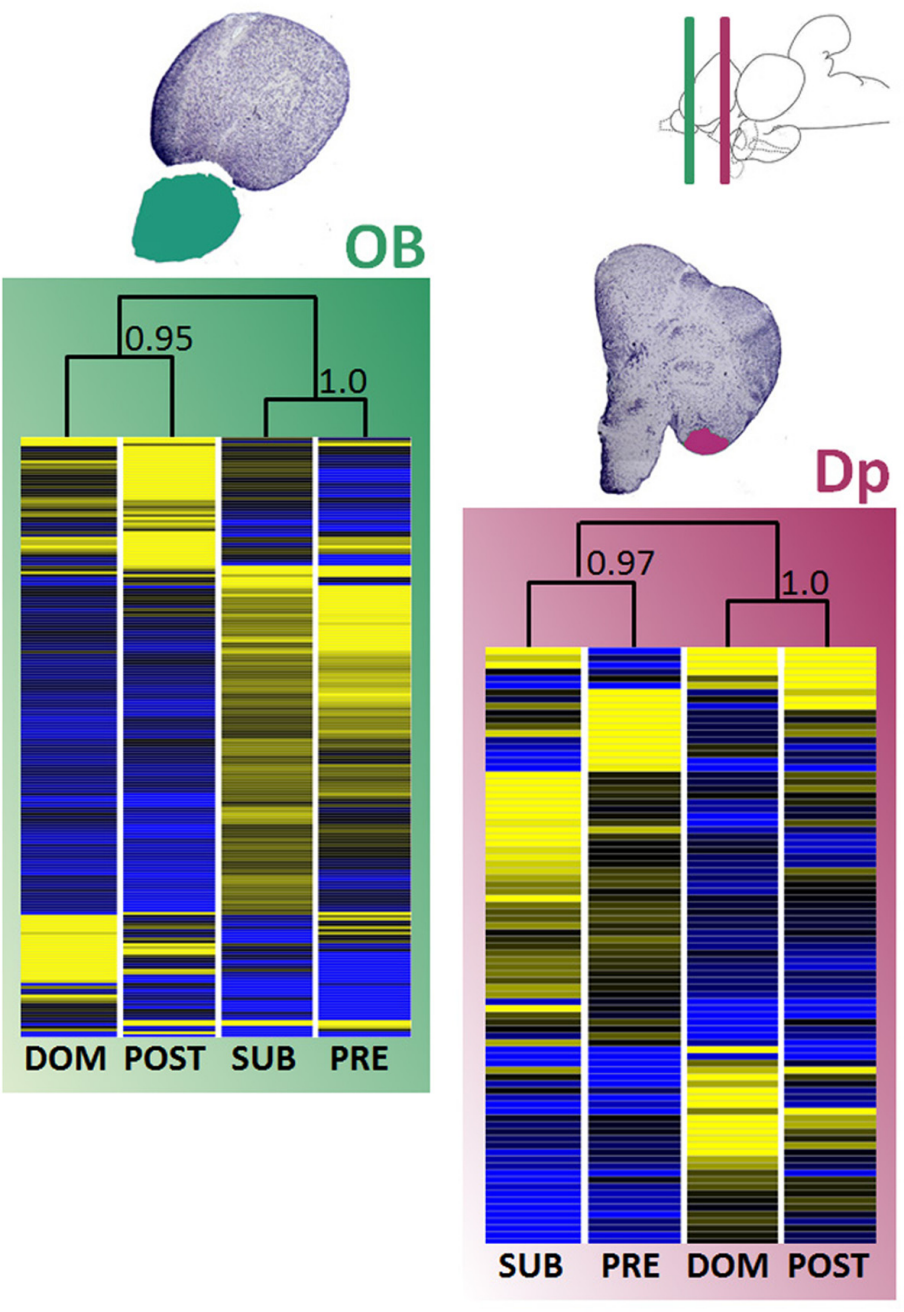
**Unsupervised hierarchical clustering of differentially expressed genes (p < 0.01) for all four olfactory stimuli and both brain areas sampled (OB and Dp).** On the top right, a sagittal view of a tilapia’s brain cut by two lines (green and violet) representing the location of the coronal cuts depicted just below illustrating the areas sampled (OB and Dp; Nissl stained slices, 10 μm). Bootstrap values are shown on clustergrams. On the heatmaps, blue represents significantly down-regulated genes, yellow up-regulated genes and black intermediate levels of expression. Confidence values of cluster nodes were calculated using bootstrapping (1000 permutations with re-sampling). Olfactory stimuli used in this study: DOM- dominant male urine; SUB- subordinate male urine; PRE- pre-ovulatory female water extract; POST- post-ovulatory female water extract. Brain regions analyzed: olfactory bulb (OB), green box; posterior part of the dorsal telencephalon (Dp), purple box.

**Figure 3:**
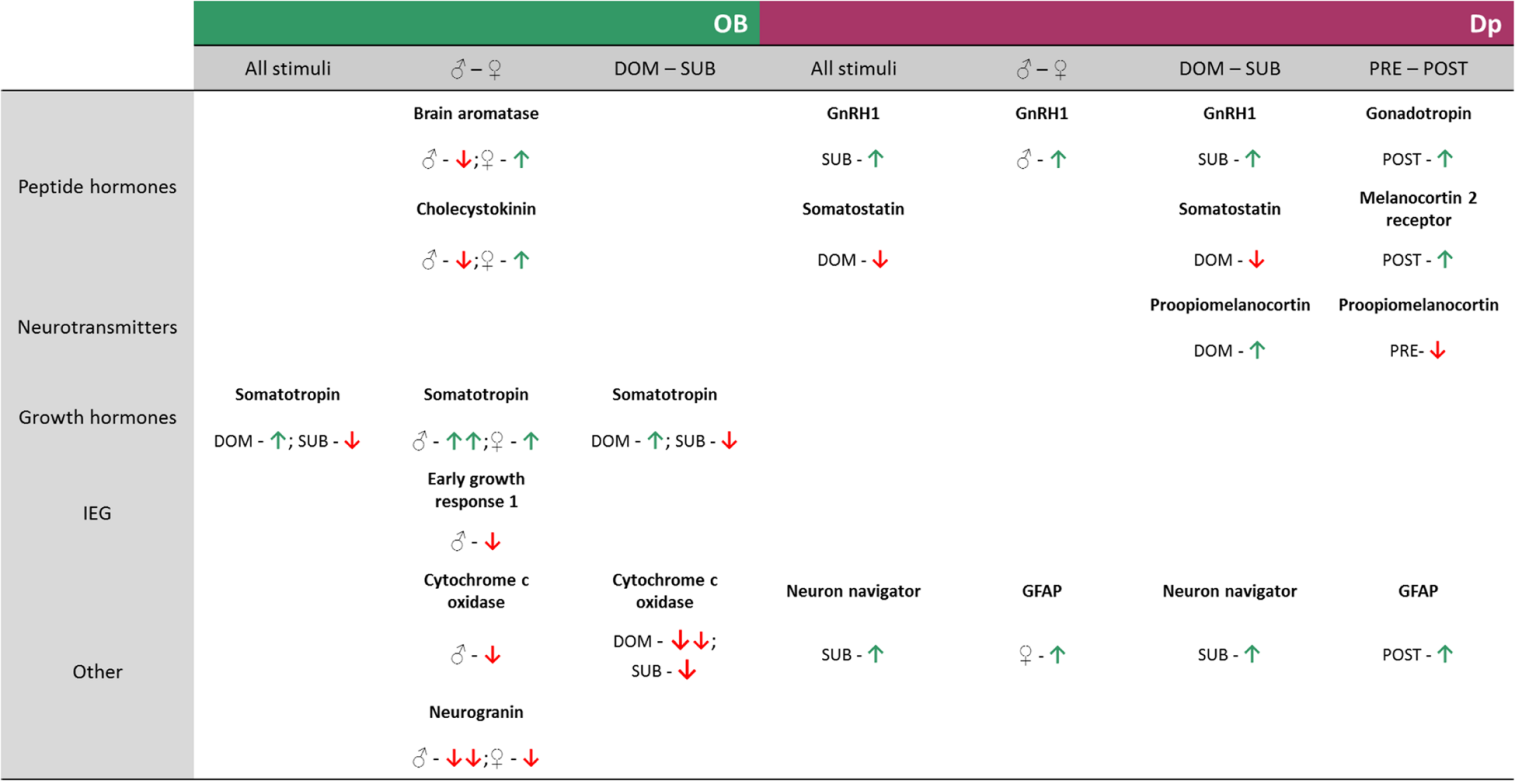
**List of all significantly regulated candidate genes for each one of the four olfactory comparisons made for both brain areas, organized according to presumed functional categories.** On the top, are represented both brain areas sampled (OB - green and Dp - violet), below are presented some significantly expressed target genes according to the chemical categories compared for each region. Green (up) and red (down) arrows indicate if genes were over- or under-expressed, respectively. Olfactory stimuli compared: DOM- dominant male urine; SUB- subordinate male urine; PRE- pre-ovulatory female water extract; POST- post-ovulatory female water extract.

Analysis of OB and Dp gene expression revealed hundreds of differently expressed genes after stimulation with any of the four different social stimuli (Table 1). Considering the initial more than 19 K unique expressed sequence tags (ESTs) included in the analysis, over 72% hybridized with our samples (i.e. presented a signal- to-noise ratio above threshold) in both OB and Dp, confirming the usefulness of heterologous hybridization [35,36]. A Bayesian analysis of gene expression levels [37] revealed that 211 of the surveyed genes in the OB showed significant differences among the four olfactory stimuli, whereas in Dp only 87 genes were differentially expressed (p < 0.01; Figure 2). No genes were found to be up- or down-regulated simultaneously in both regions, suggesting that region specific molecular processes are activated by olfactory stimulation and neural transmission. Another interesting observation concerning the number of differently expressed genes in each of these two olfactory processing centers was that at the first relay station, OB, the comparison between male and female cues seems to elicit a considerable surplus of gene regulatory activity, with more than 500 genes being differently expressed (Figure 3). However, at the olfactory pallium (Dp) this number decreases substantially and the comparison between PRE and POST females emerges with almost 200 differently expressed genes (Figure 3).

**Table 1:** **List of all significantly expressed genes and GO terms, organized by each one of the four olfactory phenotypes compared for both brain areas tested**

	**OB**			**Dp**		
**Olfactory phenotypes compared**	**Differently expressed genes**	**#features GO analysis**	**Sample sizes**	**Differently expressed genes**	**#features GO analysis**	**Sample sizes**
DOM-SUB-PRE-POST	211	118	5-6-6-6	87	52	5-4-6-5
♂ - ♀	504	271	11-12	91	66	9-11
DOM-SUB	185	109	5-6	128	75	5-4
PRE-POST	96	56	6-6	197	172	6-5

Table includes differently expressed genes, the number of features annotated considered for the Gene Ontology analysis and the sample size considered for each phenotype, each comparison and each area sampled. DOM- dominant male urine; SUB- subordinate male urine; PRE- pre-ovulatory female water extract; POST- post-ovulatory female water extract. (P < 0.01).

A hierarchical cluster of these differently expressed genes in the OB and Dp revealed interesting patterns of neuromolecular activity. In both brain regions, the transcriptional response of males exposed to DOM male urine was most similar to that of males exposed to POST female water extract, and the transcriptional response to SUB male urine was most similar to the response to PRE female water extract when we compare all stimuli (Figure 2).

The evidence for olfactory discrimination among stimuli in both brain regions reinforces the idea of a functional organization of the fish olfactory system with parallel pathways flowing from the sensory epithelia via the olfactory bulb into the pallium, conveying specific odor information [19,38]. Furthermore, the different brain regions seem to preferentially process certain stimuli, with sex differences in odors being mainly processed in the OB and subsequent odor differentiation within each sex being processed in area Dp. Cummings et al. [17] concluded that these neuromolecular processes drive behavioral responses in the context of female mate choice in swordtails. Unlike olfactory cues in our experiments, female choice in this species activated a suite of genes in response to classes of social stimuli: specific pathways were either up- or down-regulated when females were exposed to males or to other females.

From an ecological point a view, these surprisingly similar transcriptional responses of the OB and Dp to SUB males and PRE females might be explained by the distinctive information conveyed by each behavioral phenotype and by shared valence and salience of their odors. It is possible that chemical signals emitted by SUB males are feminized, which would help to explain why DOM males are occasionally observed to direct courtship behavior towards SUB males [28]. SUB males and PRE females shoal together and share the same body coloration. When courted by DOM males SUB males exhibit female-like behaviors, which include following the DOM male to the spawning pit and getting involved in the full spawning sequence [28]. This behavior allows SUB males to remain inside the breeding aggregations where they might attempt sneak fertilizations [28,39]. Despite having mature testes [40], SUB males present lower androgen levels [27], lower expression of secondary sex characters [40], and undergo androgen-dependent morphological changes in the urinary bladder and urine storage capacity, reducing its volume to a more female-like size [25], which may also affect the composition of their urine.

The similarity between the gene expression patterns elicited by DOM male and POST olfactory signals is more difficult to explain. Both social phenotypes are usually very aggressive [33,34], which might explain some similarities in chemical information. Other possible similarities of the odor bouquet released by these two groups could be related to the significantly reduced food intake these fish experience compared with SUB males and PRE females, respectively, or the high metabolic rates needed to endure a continuous effort like territorial defense by DOMs or offspring care by POST females [34,41]. In rodents, for example olfactory sensitivity seems to increase in fasted animals [42], possibly due to a leptin-based modulation of the olfactory mucosa in response to the nutritional status of the animals [43]. This link between food intake and olfaction in rodents could potentially be present in teleosts since it could act as an important eco-ethological adaptation increasing the efficacy of foraging animals when fasted.

**Figure 4:**
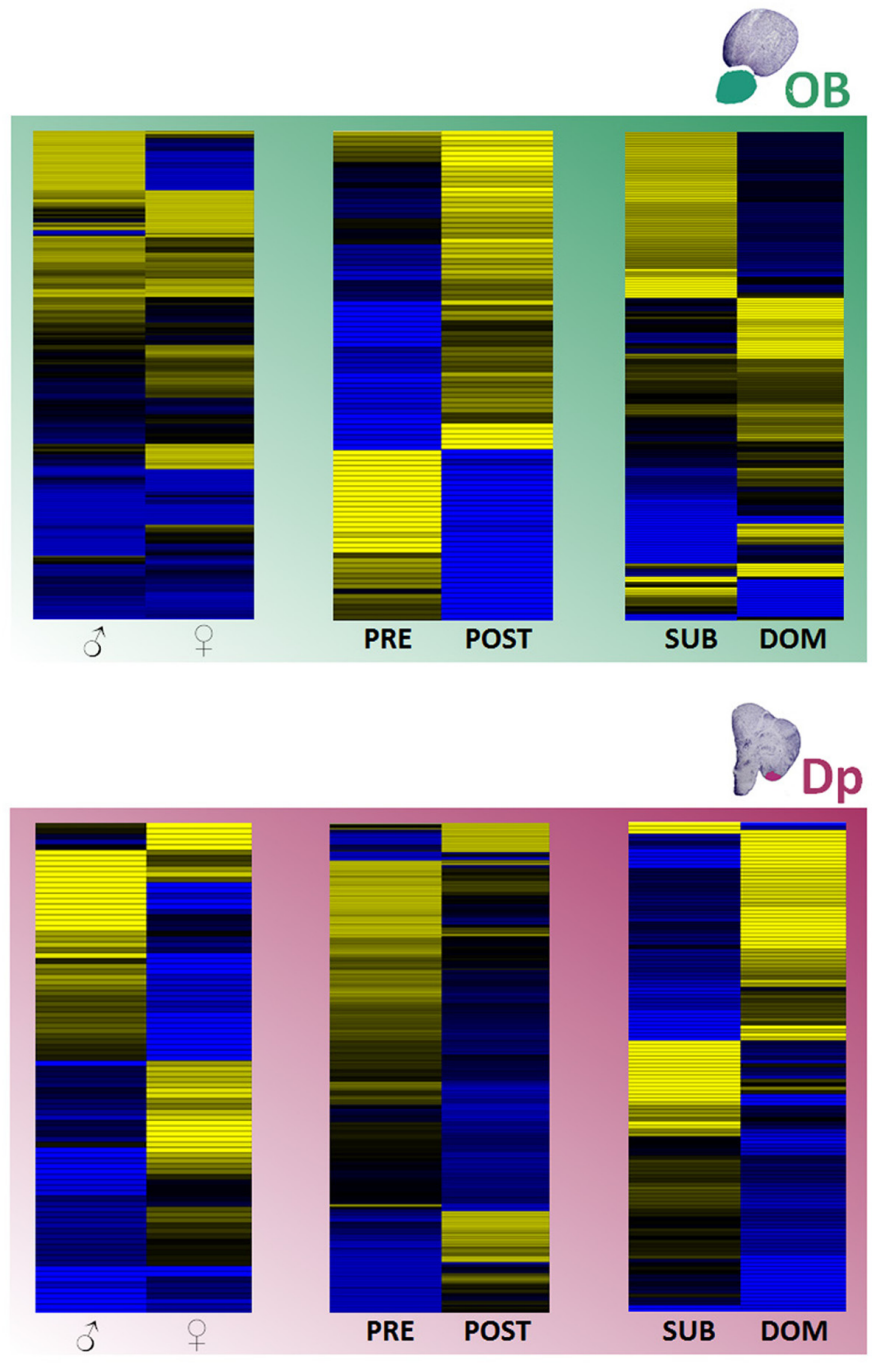
**Hierarchical clustering of significantly different expressed genes (** ***P*** **< 0.01) for the comparison of three pairs of olfactory cues in both brain areas sampled (OB and Dp).** Green box: olfactory bulb (OB) expression; purple box: posterior part of the dorsal telencephalon (Dp). Left panels: comparison of female (symbol) and male (symbol) cues independent of status or condition; middle panels, comparison of pre- (PRE) and post- (POST) ovulatory female cues; right panels: comparison of dominant and subordinate male cues. The heatmaps (blue – down-regulated, yellow – up-regulated) show estimated gene expression levels. Confidence values of cluster nodes were calculated using bootstrapping.

The comparison between transcriptional profiles of males stimulated with social olfactory cues with the electrophysiological data gathered from the same males but at the level of the olfactory epithelium also raises some interesting points. The olfactory epithelium appears to be more sensitive to DOM male and PRE female stimuli but discrimination between the sexes does not seem to occur at this level of sensory processing (Figure 1). However, at the level of the OB the gene expression profiles suggest that males have the relevant information available that allows sex discrimination (Figures 1 and 4), reinforcing the salience of olfactory cues in social communication in cichlids and teleost fishes in general [19,44]. Since EOG recordings and gene expression were collected from the same individuals, we calculated correlation coefficients between the average EOG amplitude and the expression levels of the genes that were differentially expressed across the four treatments for the two brain regions studied. Considering a r ≥ Consias indicative of an association with a high effect size [45], 14.7% (31/211) and 18.4% (16/87) of the differentially expressed genes showed high effect size correlations with the EOG response in the OB and Dp areas, respectively (see Additional file 1). The fact that mRNA levels of only a minority of genes that were differentially expressed in olfactory processing brain regions correlated with the response of the sensory epithelium might be due to the fact that the EOG is an extracellular recording that integrates the overall response of the olfactory mucosa to the stimulus. Therefore, it mainly represents the salience of the stimulus to the fish. On the other hand, the differentially expressed genes are part of specific signaling pathways that most probably were being activated in response to different dimensions of the stimulus as it was being processed at the different relay stations of the olfactory system. Therefore, the overall lack of association between EOG amplitude and gene activity is not surprising.

### GO analysis

**Figure 5:**
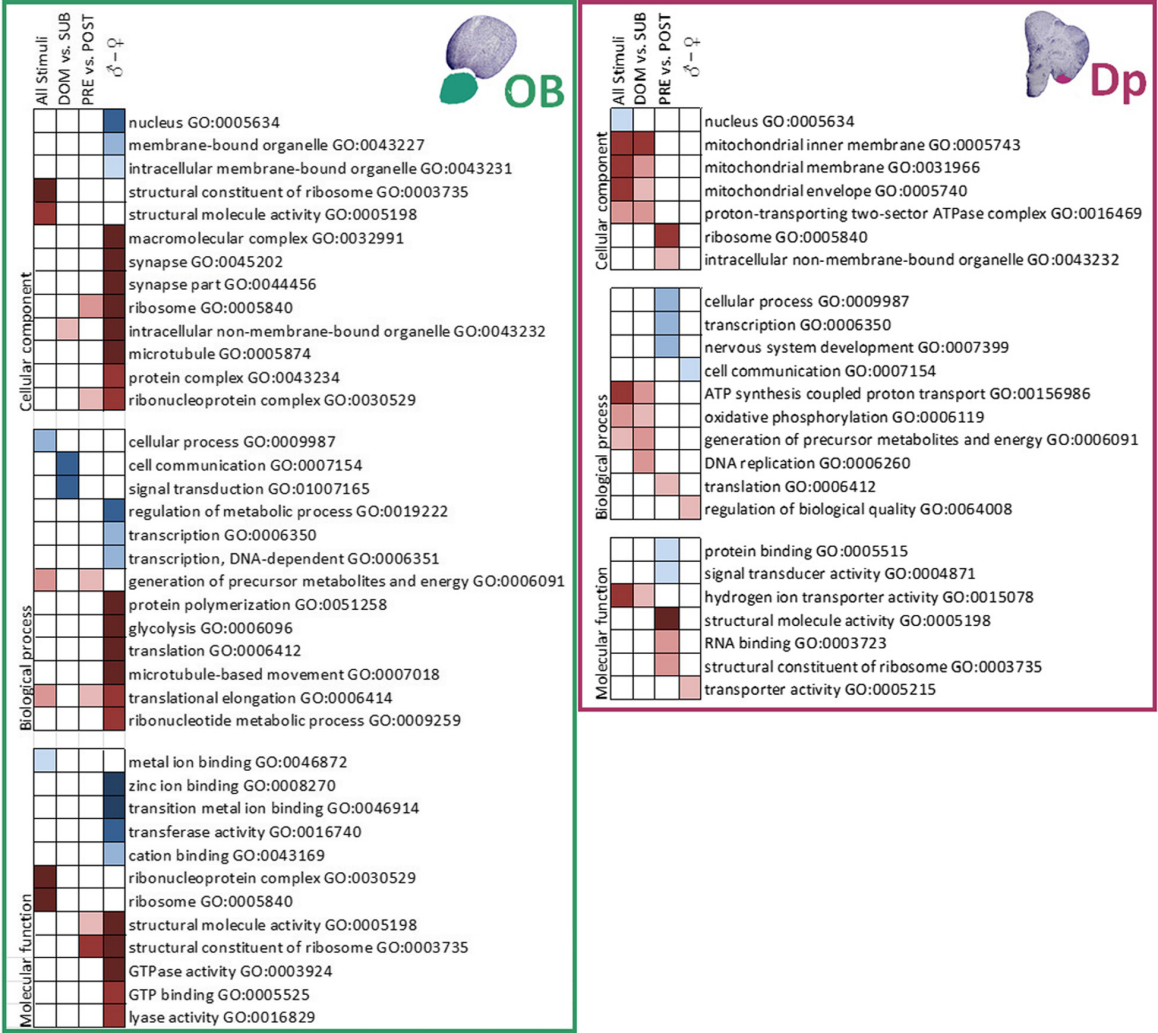
**Gene Ontology (GO) analysis summary for each one of the four olfactory comparisons made for both brain areas.** Under-represented categories are shown in shades of blue and over-represented ones in shades of red (from p < 0.05 to p < 0.001). The different GO vocabularies are shown separately: cellular component, biological process and molecular function; along with the *P*-values (uncorrected results of the hypergeometric test) and GO names and numbers (according to 200605 releases). DOM- dominant male urine; SUB- subordinate male urine; PRE- pre-ovulatory female water extract; POST- post-ovulatory female water extract.

Our Gene Ontology analysis allowed us to categorize differentially expressed genes in relation to the molecular functions, biological processes, and cellular components they are associated with (Figure 5). Although the results of GO analyses can be difficult to interpret, they provide a framework for developing novel hypotheses that could potentially inform new approaches to the molecular underpinnings of socially regulated brain function [8]. We therefore asked whether any GO categories are under- or over-represented in one stimulus condition compared to the others. In all comparisons analyzed (DOM male vs. SUB male vs. PRE female vs. POST female odors; male vs. female odors; PRE vs. POST female odors; and DOM male vs. SUB male odors), GO terms could be applied to more than 55% of the regulated array features. Interestingly, the functional categories expressing enriched pathways with extreme over- and under-representation are also more numerous for the distinction between males and females in the OB, and rather scarce for the same comparison at the Dp level. In the latter, the number of enriched GO terms is smaller and more evenly distributed among the remaining comparisons (DOM vs. SUB male odor and PRE vs. POST female odor). This suggests that already at the OB level, the first relay station in the olfactory circuit, information on the sex of a nearby conspecific might be filtered out, which in a social interaction would be reinforced by visual cues ascertaining this information and triggering the appropriate behavioral response.

### Candidate genes

Besides activating specific molecular pathways, a number of candidate genes are also significantly regulated in one of the two brain areas sampled from dominant males stimulated with different social odors in this experiment. Somatotropin, a member of the Growth Hormone (GH) family, is significantly up-regulated in the OB (Figure 3) in response to either the odor of a DOM male or the odor of females (either PRE or POST). On the other hand, in Dp somatostatin, a known GH production inhibitor, is down-regulated after stimulation with DOM scent. Regulation of these members of the GH signaling are usually related to differential growth, a characteristically plastic trait in cichlids in response to changes in the social environment [46]. Somatostatin is known to play an important role in the complex interplay between social behavior and somatic growth in cichlid fishes [47], likely regulating the allocation of energetic resources between reproduction and growth [48]. Somatostatin down-regulation only in response to the presence of an odor cue of a potentially threatening high-ranked male along with the up-regulation of somatotropin, suggests the preparation for the physical strain involved in an approaching agonistic interaction.

Other candidate genes were also up-regulated in stimulated dominant males, such as: brain aromatase in the OB and gonadotropin-releasing hormone (GnRH1) and pro-opiomelanocortin alpha 2 (*pomc α2*) in Dp. It has been previously showed in *A. burtoni* that *pomc α2* is more highly expressed in the brain of DOM males compared with SUBs [8], and α-melanocyte stimulating hormone (one of the principal peptides encoded by *pomc*) has been shown to stimulate DOM traits such as aggression and coloration [49]. Interestingly, *pomc α2* exhibits a complex expression pattern through brain and peripheral tissues of in *A. burtoni* [4], which makes this gene a prime candidate for future studies. The up-regulation of GnRH1 after an olfactory stimulation with SUB male odor reinforces the idea of a putative feminization of their urine discussed above, since GnRH integrates the animal’s internal physiological state with incoming external cues to regulate reproduction in males. In cichlid fish, reproductive status influences the regulation of this neuropeptide and seasonal fluctuations of GnRH receptor levels in the brain can modulate olfactory processing, regulating the animal’s plasticity in olfactory responsiveness [50]. Importantly, GnRH receptors are expressed in both OB and Dp of the Nile tilapia, *Oreochromis niloticus* [51,52]. Interestingly, GnRH up-regulation in an extra-hypothalamic area, like Dp, can also be found in rats, where GnRH mRNA is present both in the olfactory piriform cortex (homolog of Dp) and in the olfactory bulb [53].

*egr-1* and cytochrome C oxidase (COx) were both down-regulated in OB of males stimulated with male social odor when compared with female social odor. Both genes are known markers of neural activity [54,55] and the regulation of *egr-1* appears to have a pivotal role in recruiting specific neural pathways required for long-term memory processes [54]. *egr-1*-deficient mice seem to be unable to form long-term memories in behavioral tasks, such as olfactory discrimination, while their short-term memory and early-LTP remain intact [56]. In zebrafish, *egr-1* activity seems to be involved in imprinting processes in early life stages and later in kin recognition, especially in the OB, since rather low basal expression levels are found in the Dp [57]. In summary, *egr-1* down-regulation in the OB of DOM males in response to olfactory cues of male conspecifics, suggests a possible role of olfactory modulation on memory consolidation of social odors, but on the other hand, little is known about COx modulation with olfactory social stimuli. In another cichlid species, *Astatotilapia burtoni*, when males were presented with visual and olfactory signals, each sensory modality was sufficient to elicit an androgen response in an intruder challenge paradigm, but chemical stimulation alone did not induce the immediate-early gene c-Fos, another marker of neuronal activity [58], in the brain [18].

## Conclusions

In the present study we have used a transcriptome-scale analysis of the molecular systems regulated by social olfactory experience in order to investigate the proximate mechanisms underlying olfactory stimulation. We found that DOM males stimulated with different socially salient chemical cues exhibited some degree of discrimination between stimuli in the olfactory epithelium. Also, different salient olfactory stimuli resulted in considerable variation in OB and Dp gene expression profiles of DOM males, suggesting that the olfactory system can discriminate social status and reproductive condition, as well as, its sex based solely on its chemical signature. Our findings also underscore the extensive transcriptional plasticity in response to the social environment and reinforce the importance of uncovering the molecular and cellular factors and constraints governing olfactory function and the neurogenomic consequences of experiencing different social olfactory cues. These different neurogenomic states likely modulate and optimize behavior according to social context [59]. Future comparative studies focusing on the neuroplasticity underlying the diverse behavioral adaptations found in cichlids will help us understand the processes by which this teleost family has diversified so rapidly.

## Methods

### Housing

Mozambique tilapia used for stimuli collection were housed at ISPA – Instituto Universitário, Lisboa, Portugal in mixed-sex groups which were kept in tanks with gravel substrate, which promotes nest digging by males and the establishment of territories and social hierarchies, at a temperature of 26 ± 2°C and a 12 L:12D photoperiod. Mozambique tilapia used for the microarray analysis were obtained from a brood-stock (inbred line) maintained at the University of Algarve (Faro, Portugal), which derived, like the one maintained at ISPA-IU, from the Vasco da Gama Aquarium stock, originated from individuals collected at Incomati River (Mozambique) in the early 1970s. Since all specimens examined are mixed siblings deriving from an inbred line, the potential impact of genetic relatedness on our transcriptome data is negligible. Fish were fed twice daily with commercial cichlid sticks.

### Chemical stimuli

In different tanks, stable social groups of 10 individuals (5 males and 5 females) were left undisturbed for 5 to 8 weeks. During this period, territories were established and spawning occurred naturally. Five minute behavioral observations of each individual were done every other day and male social status and behavior was noted.

Different sampling approaches were used to collect social odors for each sex due to the intrinsic biological differences between them. Given that male tilapia store urine in their bladders, urine was collected in males by a smooth anterior-posterior massage of the abdominal region following a procedure previously described [27]. Urine from three males was pooled according to social status (DOM or SUB). Since it is very difficult to collect urine from females, female-conditioned water was used instead. For this purpose females were isolated in 20-L glass tanks with dechlorinated tap water (at 27°C) for 4 h (according to [26]). This conditioned water was divided in two groups of three females each, designated as either PRE or POST, depending on the sampling point being either the day prior to their predicted ovulation day or 1–2 days after they have spawned, respectively. Female reproductive stage was determined by systematic observations of their behavior, abdomen profile and genital papilla. All samples (both female conditioned-water samples and male urine samples) were then subjected to a fractionation procedure similar to the one described in [60].

### Electro-olfactogram (EOG) and brain microdissection

In order to characterize the responses elicited by the stimuli used in this experiment, EOGs were recorded in 33 dominant male tilapia (body mass = 182 ± 34 g) using a similar protocol to that described in [26]. Briefly, each male was anaesthetized by immersion in water containing 100 mg l^−1^ MS-222 (Pharmaq, Norway) and immobilized with an intramuscular injection of gallamine triethiodide (3 mg kg^−1^ in 0.9% saline). Immobilized fish were then placed in a purpose-built V-clamp and aerated, via a mouthpiece, with water containing 50 mg l^−1^ MS-222. The right-side olfactory rosette was exposed by removal of the ring of cartilage surrounding the nostril and continuously irrigated with dechlorinated, charcoal-filtered water via a gravity-fed system (6 ml min^−1^). The EOG was recorded using the software Axoscope (Axon Instruments, Inc., Foster City, CA, USA). The peak amplitude of the EOG was measured, blank-subtracted and normalized (using the response to the ‘standard’ 10^−5^ mol l^−1^ L-serine) as described by [60]; blanks and standards were run twice, in the beginning and end of the recording period for each replicate. This normalization of the EOG amplitudes was to reduce variability caused by small differences in electrode positioning, and/or in the olfactory sensitivity within (during the stimulation period) and among fishes.

Each fish was exposed to a single olfactory stimulus, introduced into the continuous water flow via a three-way valve, for 5 s with 10 s intervals for a period of 45 min. This frequency of stimulation allowed for olfactory neurons to return to a baseline state before the next stimulation; also a pulsatile olfactory stimulation reflects the rate of urine pulses by males during social interactions [21,26]. In sum, tilapia males were stimulated with blank, dominant and subordinate stimuli (N = 7 per group) using the protocol described above, while the remaining were stimulated with female odors (N = 6 per group). After the olfactory stimulation, males were killed by decapitation within 2 minutes and the brains were rapidly dissected, embedded in Tissue-Tek® OCT™ Compound, and stored at −80°C. That same day and always the same researcher sliced the fish brains (sectioned into 200 μm transverse slices) in a temperature-controlled (−18°C) cryostat. The olfactory bulbs (OB) and the putative olfactory pallium (area Dp – posterior part of the dorsal telencephalon) were then microdissected from the appropriate sections using a 27G gauge micropunch cannula [61]. The number of fish tested per day was limited in order to allow the stimulation procedure to be always conducted during the same part of the day (i.e. 10 h-12 h).

### Microarray analysis

Total RNA was extracted from both microdissected brain areas (OB and Dp) according to a standard Trizol protocol (Invitrogen) and subjected to one round of RNA amplification using Message Amp II kit (Invitrogen). Amplified RNA was analyzed for quantity and quality on the Bioanalyzer 2100 (Agilent) using the Agilent Total RNA Nano Chip assay. Samples from blank stimulations (control) collected from seven different individuals (for both areas) were pooled and aliquoted to be used as common reference in a reference based array design (see Table 1 and Additional file 2). In this design the intensity of hybridization to a given spot for a sample of interest (i.e. DOM, SUB, PRE or POST) is measured relative to the intensity of hybridization of the same spot on the same array but for the reference sample (i.e. pool of blank stimulations). mRNA (500 ng) from each experimental sample or reference were reverse transcribed using SuperScript II (Invitrogen) and labeled according to [35]. Following this reverse transcription, RNA was hydrolyzed and purified before being dye-coupled with Cy3 or Cy5 post-labeling Reactive Dye Pack (Amersham). A reference and experimental sample were competitively hybridized at 65°C overnight to a 19 K *A. burtoni* cDNA microarray (GEO platform GPL6416) constructed from brain-specific and mixed tissue libraries representing a total of 17,712 cichlid-specific features [35,62]. This platform has previously been shown to give biologically meaningful results in heterologous hybridizations using *Oreochromis sp.* [35]. Finally, microarrays were scanned with an Axon 4000B scanner (Axon Instruments) using Genepix 4.0 software (Axon Instruments). Array features were visually inspected individually and features with poor quality, that is, with a signal intensity smaller than twice the standard deviation above background, or displaying irregularities or potentially erroneous artifacts were excluded.

### Statistical analysis

Normalized EOG amplitudes in response to DOM, SUB, POST and PRE stimuli were compared by a one-way ANOVA followed by the least significant difference (LSD) test for comparison between two mean using the software package STATISTICA v.10 (StatSoft, Inc., 2011).

Microarray data were processed using the LIMMA software package (v3.12.0; [63] in R (v2.15.0; the R Foundation for Statistical Computing, 2012)). Background-subtracted mean intensities were calculated using the minimum method and further normalized using within-array loess normalization. After this normalization step, Bayesian analysis was used to calculate gene expression levels using the ratios of intensities measured. Finally, to compare between expression profiles for the different olfactory stimulations, unsupervised hierarchical clustering analysis were done using the *hclust* function in R/Bioconductor. The reference-based design allows the comparison of any subset of samples to any other subset using cluster analysis since the relative expression measurements are consistent with regard to the same reference. Thus, we compared not only all subsets of samples (all stimuli analysis, Figure 2) to ascertain similarities and differences in transcriptome regulation between all olfactory stimuli. We also wanted to understand whether individuals responded differently to male (DOM and SUB) or female stimuli (PRE and POST; Figure 4). Finally, we compared the subset of male and female stimuli to evaluate whether in either of the two candidate regions the genomic responses to the two male and female phenotypes would be different (Figure 4). The *heatmap* function in the package *gplots* was used to visualize clusters of gene expression, where only significantly expressed genes (P < 0.01) across conditions were clustered. The consensus tree and confidence values were calculated via bootstrapping datasets, based on the Euclidian distanced matrix obtained for each of the 1000 permuted gene expression profile datasets.

Regarding the functional annotation of ESTs, we considered a library already compiled for another cichlid species, *A. burtoni*, and used Cytoscape (v.2.8, [64]) with the BiNGO plug-in (Biological Network Gene Ontology tool, [65]) for the calculation of under- and over-represented GO terms considering our larger data set (comparison between all stimuli: DOM vs. SUB vs. PRE vs. POST) and reported uncorrected hypergeometric *p*-values.

### Availability of supporting data

The raw and analyzed data for the 43 microarray experiments used in this study have been submitted to Gene Expression Omnibus (SERIES ID = GSE54468, available online http://www.ncbi.nlm.nih.gov/geo/query/acc.cgi?acc=GSE54468). The ESTs representing the cDNAs on the microarray have been submitted to NCBI GenBank.

### Ethics

This study was performed in strict accordance with the recommendations of the Direcção Geral de Veterinária, the Portuguese National Authority for Animal Health, and the protocol was approved by their ethics committee (Permit Number: 0420/000/000/2007). All surgery was performed under MS222 anesthesia, and every effort was made to minimize suffering.

## Additional files

Additional file 1:
**List of genes expressing correlations with the average EOG amplitude (across the 4 treatments) for the two brain regions studied (OB and Dp).** Only associations with a high effect size (r ≥ 0.5) are presented. Additional file 2:
**Hybridization design of control and reference samples.** Brain samples from blank stimulations (control) collected from seven different individuals (for both areas) were pooled and aliquoted to be used as reference in a reference based array design. mRNA (500 ng) from each experimental sample or reference were reverse transcribed and RNA was hydrolyzed and purified before being dye-coupled with Cy3 or Cy5. A reference and experimental sample were competitively hybridized overnight.

## Abbreviations

DOM: Dominant male
SUB: Subordinate male
PRE: Pre-ovulatory female
POST: Post-ovulatory female
EST: Expressed sequence tag
GO: Gene ontology
OB: Olfactory bulb
Dp: Posterior portion of the dorsal telencephalon
GnRH1: Gonadotropin-releasing hormone (GnRH1)
*pomc α2*: Pro-opiomelanocortin alpha 2
