# Supplementary figures and images for: Social odors conveying dominance and reproductive information induce rapid physiological and neuromolecular changes in a cichlid fish
Source: BMC Genomics. 2015 Feb 22;16(1):114. doi: 10.1186/s12864-015-1255-4 (PMC4344806; doi:10.1186/s12864-015-1255-4)

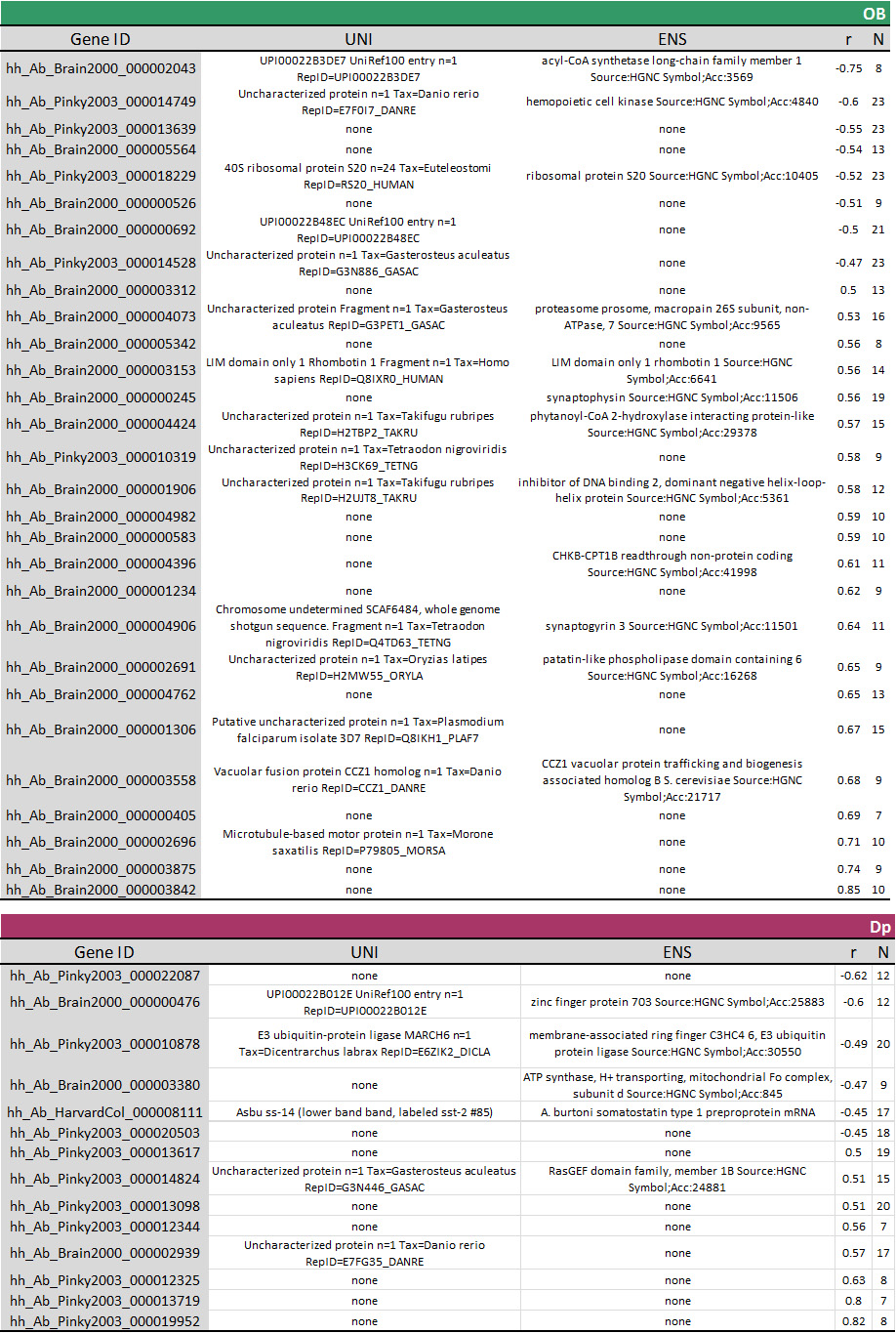

Supplement: Additional file 1: — List of genes expressing correlations with the average EOG amplitude (across the 4 treatments) for the two brain regions studied (OB and Dp). Only associations with a high effect size (r ≥ 0.5) are presented. [file 12864_2015_1255_MOESM1_ESM.jpeg]

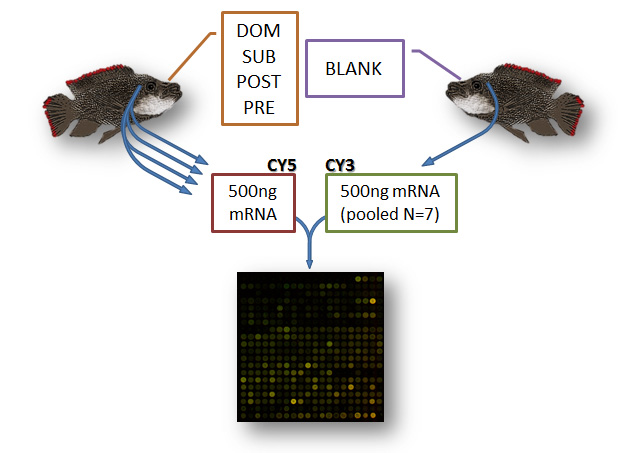

Supplement: Additional file 2: — Hybridization design of control and reference samples. Brain samples from blank stimulations (control) collected from seven different individuals (for both areas) were pooled and aliquoted to be used as reference in a reference based array design. mRNA (500 ng) from each experimental sample or reference were reverse transcribed and RNA was hydrolyzed and purified before being dye-coupled with Cy3 or Cy5. A reference and experimental sample were competitively hybridized overnight. [file 12864_2015_1255_MOESM2_ESM.jpeg]
